# Structural and phylogenetic analyses of resistance to next-generation aminoglycosides conferred by AAC(2′) enzymes

**DOI:** 10.1038/s41598-021-89446-3

**Published:** 2021-06-02

**Authors:** Angelia V. Bassenden, Linda Dumalo, Jaeok Park, Jonathan Blanchet, Krishnagopal Maiti, Dev P. Arya, Albert M. Berghuis

**Affiliations:** 1 Department of Biochemistry, McGill University, McIntyre Medical Building, 3655 Promenade Sir William Osler, Montreal, QC H3G 1Y6 Canada; 2 Centre de Recherche en Biologie Structurale, McGill University, Bellini Life Science Complex, 3649 Promenade Sir William Osler, Montreal, QC H3G 0B1 Canada; 3 Department of Chemistry, Clemson University, Clemson, SC 29634 USA; 4 Department of Microbiology and Immunology, McGill University, Duff Medical Building, 3775 University Street, Montreal, QC H3A 2B4 Canada

**Keywords:** Structural biology, X-ray crystallography, Biochemistry, Transferases

## Abstract

Plazomicin is currently the only next-generation aminoglycoside approved for clinical use that has the potential of evading the effects of widespread enzymatic resistance factors. However, plazomicin is still susceptible to the action of the resistance enzyme AAC(2′)-Ia from *Providencia stuartii*. As the clinical use of plazomicin begins to increase, the spread of resistance factors will undoubtedly accelerate, rendering this aminoglycoside increasingly obsolete. Understanding resistance to plazomicin is an important step to ensure this aminoglycoside remains a viable treatment option for the foreseeable future. Here, we present three crystal structures of AAC(2′)-Ia from *P. stuartii*, two in complex with acetylated aminoglycosides tobramycin and netilmicin, and one in complex with a non-substrate aminoglycoside, amikacin. Together, with our previously reported AAC(2′)-Ia-acetylated plazomicin complex, these structures outline AAC(2′)-Ia’s specificity for a wide range of aminoglycosides. Additionally, our survey of AAC(2′)-I homologues highlights the conservation of residues predicted to be involved in aminoglycoside binding, and identifies the presence of plasmid-encoded enzymes in environmental strains that confer resistance to the latest next-generation aminoglycoside. These results forecast the likely spread of plazomicin resistance and highlight the urgency for advancements in next-generation aminoglycoside design.

## Introduction

The discovery of the first aminoglycoside, streptomycin, revolutionized antibiotic usage, being the first useful bacterial-sourced antibiotic to offer effective treatment for tuberculosis^1,2^. This led to the rapid succession of novel aminoglycoside antibiotic discovery over a 30 year period, which largely masked the emergence of concomitant aminoglycoside resistance^3^. Their range of biological activity and medical applications, despite their toxicity, made them an essential tool in the fight against serious bacterial infections^1,3,4^. However, at present time widespread aminoglycoside resistance has curtailed usage, where aminoglycoside modifying enzymes (AMEs) are the most significant factors attributed to antibiotic inactivation^5,6^.

Attempts to circumvent the effects of these enzymes have been numerous, including the development of semi-synthetic derivatives of naturally-occurring aminoglycosides^7^. Some of the first iterations of semisynthetic aminoglycosides, including amikacin, netilmicin, and isepamicin, introduced bulky extensions to the N1-position of kanamycin, sisomicin, and gentamicin B, respectively (see Supplementary Fig. S1 online)^7,8^. These antibiotics would prove to be more therapeutically viable than their precursor by eluding the effects of some AMEs through their inability to bind to these resistance enzymes, though they maintained affinity for their target, the 16S ribosomal A site^7^. For example, the addition of an (S)-4-amino-2-hydroxybutyrate (HABA) group to kanamycin, forming amikacin, reduced its susceptibility to AMEs by 40%^9^. Current discovery and development of next-generation aminoglycosides continue to incorporate this strategy, in combination with analyzing the structural basis for antibiotic binding^10^. Notably, these types of analyses have revealed that aminoglycosides bind to AMEs and the ribosome in their lowest energy conformation with little changes to the antibiotic, utilizing similar motifs for hydrogen bonding, while significantly varying in their van der Waals surface interactions^9,11,12^. Together, these techniques can pinpoint key features of aminoglycoside design that can allow them to evade the action of AME’s that their parent compounds were previously susceptible to.

Plazomicin, the latest next-generation aminoglycoside, was approved for clinical use by the U.S. Food and Drug Administration in 2018 and is the second semisynthetic derivative of sisomicin^10,13^. The first, netilmicin, incorporated an ethyl group at the N1 position. This modification reduced netilmicin’s sensitivity to nucleotidyltransferases, specifically, ANT(2″); however, netilmicin remained ineffective against 87% of AMEs affecting sisomicin^9^. Contrarily, plazomicin was designed to target specific pathogens which are resistant to older aminoglycosides by introducing key structural features to sisomicin^10^. As with amikacin, the addition of an (S)-HABA group at the N1 position shields plazomicin from action by phosphotransferases [APH(2″)], nucleotidyltransferases [ANT(2″)], and acetyltransferases [AAC(3)]^9,10,14^. The second substitution, a hydroxyethyl group at the 6′ position, blocks plazomicin from the activity of the most clinically prevalent and widespread enzyme group, AAC(6′) (see Supplementary Fig. S1 online)^14^. These additions to the sisomicin aminoglycoside skeleton have reduced the number of enzymes able to selectively bind and modify plazomicin to just one, AAC(2′)-Ia^14,15^.

AAC(2′)-Ia is chromosomally restricted in *Providencia stuartii*, an opportunistic pathogen responsible for catheter-associated urinary tract infections with a high mortality rate^15–17^. This enzyme is a part of a larger group of AAC(2′)-I isozymes, which includes five enzyme subtypes (AAC(2′)-Ia—AAC(2′)-Ie). Specifically, AAC(2′)-Ia utilizes cosubstrate, acetyl-CoA, to detoxify naturally occurring aminoglycosides such as kanamycin, tobramycin, sisomicin, and gentamicin, as well as semisynthetic aminoglycosides such as dibekacin, netilmicin, and plazomicin through the addition of an acetyl group at their 2′ positions^9^. Although AAC(2′)-Ia has not been a predominant factor in antimicrobial resistance in the past, the increased use of plazomicin may expand its clinical prevalence. Understanding the structural basis for this enzyme’s antibiotic selectivity is a key component in keeping plazomicin a viable treatment option for the foreseeable future.

Here, we present crystal structures of AAC(2′)-Ia from *P. stuartii* in ternary complex with naturally occurring and semisynthetic aminoglycosides: tobramycin, amikacin, and netilmicin. Together with our recently reported structure of the enzyme in complex with the next-generation aminoglycoside, plazomicin^18^, these structures have allowed us to inform the critical residues responsible for AAC(2′)-Ia′s ability to bind and modify a wide range of chemically unique 4,6-disubstituted aminoglycosides. Moreover, we present a comprehensive sequence analysis of the AAC(2′)-I homologues, outlining the conservation of residues involved in plazomicin binding across multiple bacterial species. Finally, we assess plasmid-encoded sequences identified in this analysis with high similarity and identity to AAC(2′)-Ia for their potential to modify plazomicin and rapidly spread resistance.

## Results

### Structure characteristics

**Figure 1:**
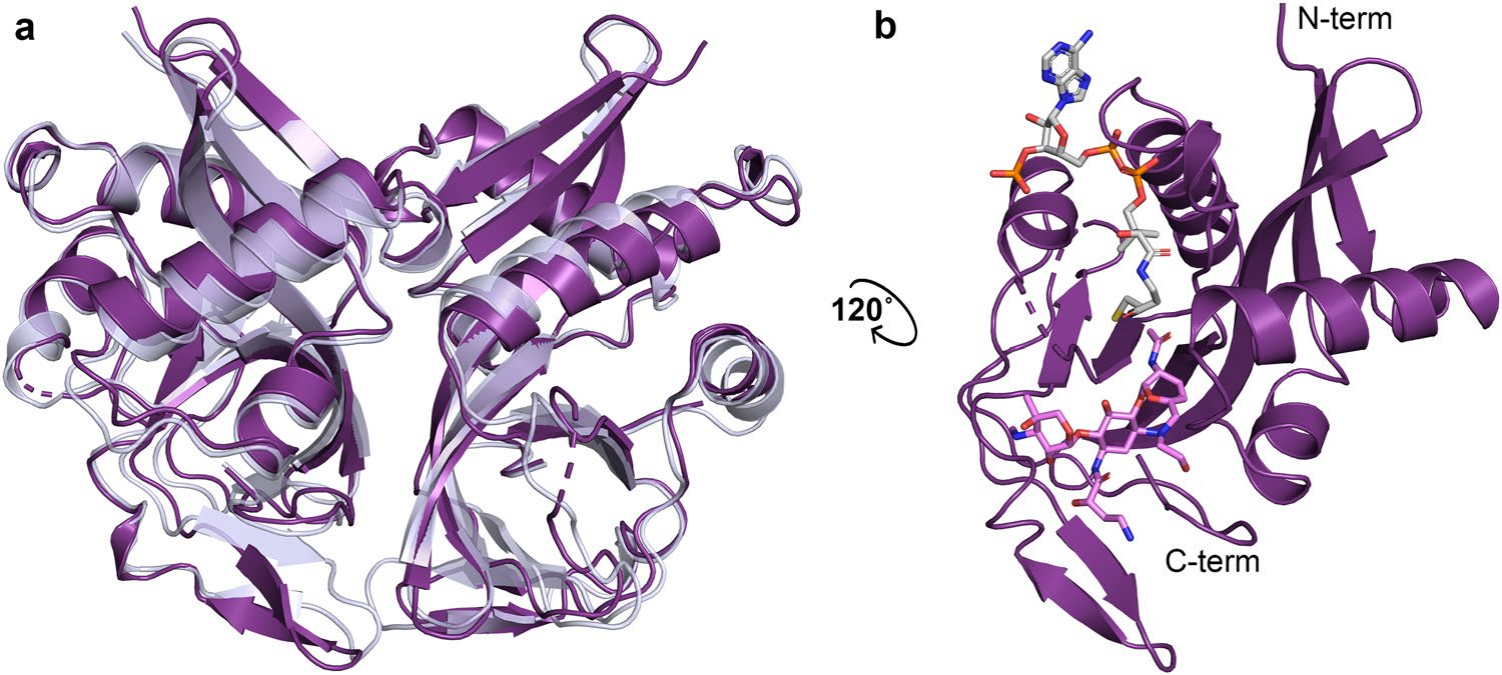
Structural features of AAC(2′)-I enzymes. (a) Overlay of AAC(2′)-Ia from *P. stuartii* colored in purple and AAC(2′)-Ic from *M. tuberculosis* (PDB ID: 1M44) colored in pale blue. (b) Monomeric unit of AAC(2′)-Ia highlighting N-terminus, C-terminus, and active site configuration, with acetylated plazomicin in violet and CoA in grey.

Three high-resolution crystal structures of AAC(2′)-Ia from *P. stuartii* were solved, two structures containing coenzyme A (CoA) and N2′-acetylated aminoglycosides tobramycin and netilmicin; and one containing acetyl-CoA and the non-substrate aminoglycoside amikacin, which lacks an N2′ moiety. Data collection and final refinement statistics for each of these crystal structures are listed in Table 1. We also recently reported the crystal structure of AAC(2′)-Ia in complex with N2′-acetylated plazomicin and CoA, and have included it here in our analyses^18^. The overall fold of AAC(2′)-Ia, as with all other reported structures of aminoglycoside N-acetyltransferases^19–29^, belongs to the GCN5 related N-acetyltransferase (GNAT) superfamily. Its dimeric structure has an identical fold to another enzyme in this subclass, AAC(2′)-Ic from *Mycobacterium tuberculosis*, which shares a 55% sequence similarity and 32% sequence identity to AAC(2′)-Ia (Fig. 1a)^19^. The overall structural features of AAC(2′)-Ia are highlighted in Fig. 1b.

**Table 1:** Data Collection and Refinement Statistics of Aminoglycoside-AAC(2′)-Ia Complexes. Statistics for the highest resolution shell are shown in parentheses.

	AAC(2′)-Ia • Acetyl-CoA • Amikacin	AAC(2′)-Ia • CoA • Acetylated-Tobramycin	AAC(2′)-Ia • CoA • Acetylated-Netilmicin
**Data collection statistics**			
Resolution range (Å)	27.47–1.42 (1.47–1.42)	26.47–1.77 (1.833–1.77)	26.44–2.0 (2.071–2.0)
Space group	C 1 2 1	P 32 2 1	P 32 2 1
Unit cell (Å, °)	93.3 58.9 83.2, β = 116.0	72.7 72.7 146.3	73.0 73.0 145.6
Total reflections	153,279 (15,254)	88,897 (8600)	62,260 (6120)
Unique reflections	76,642 (7629)	44,518 (4369)	31,130 (3059)
Multiplicity	10.3 (7.1)	7.7 (5.4)	10 (6.3)
Completeness (%)	99.4 (98.2)	99.9 (99.4)	99.9 (99.9)
Mean I/sigma(I)	21.9 (2.4)	22.7 (2.5)	20.8 (2.3)
Wilson B-factor	10.1	17.7	24.1
R-merge	0.045 (0.58)	0.025 (0.35)	0.035 (0.37)
R-meas	0.063 (0.82)	0.035 (0.50)	0.049 (0.52)
CC_1/2_	1.00 (0.48)	1.00 (0.65)	1.00 (0.72)
CC*	1.00 (0.81)	1.00 (0.89)	1.00 (0.91)
**Refinement statistics**			
R_work_	0.140 (0.284)	0.167 (0.305)	0.182 (0.301)
R_free_^a^	0.182 (0.324)	0.193 (0.290)	0.226 (0.339)
Number of non-hydrogen atoms	3809	3427	3147
macromolecules	3147	2909	2809
ligands	182	194	168
water	480	324	170
Protein residues	361	353	352
RMS (bonds)	0.005	0.006	0.006
RMS (angles)	0.78	0.72	0.79
Ramachandran favored (%)	98.0	97.7	96.5
Ramachandran outliers (%)	0	0	0
Clashscore	2.61	1.67	5.09
Average B-factor	15.9	24.7	31.5
Macromolecules	13.5	23.4	31.0
Ligands	17.0	30.4	36.2
Solvent	31.1	33.4	35.2

^a^R_free_ was calculated by randomly omitting 10% of observed reflections from refinement.

### Acetyl coenzyme A and coenzyme A binding

**Figure 2:**
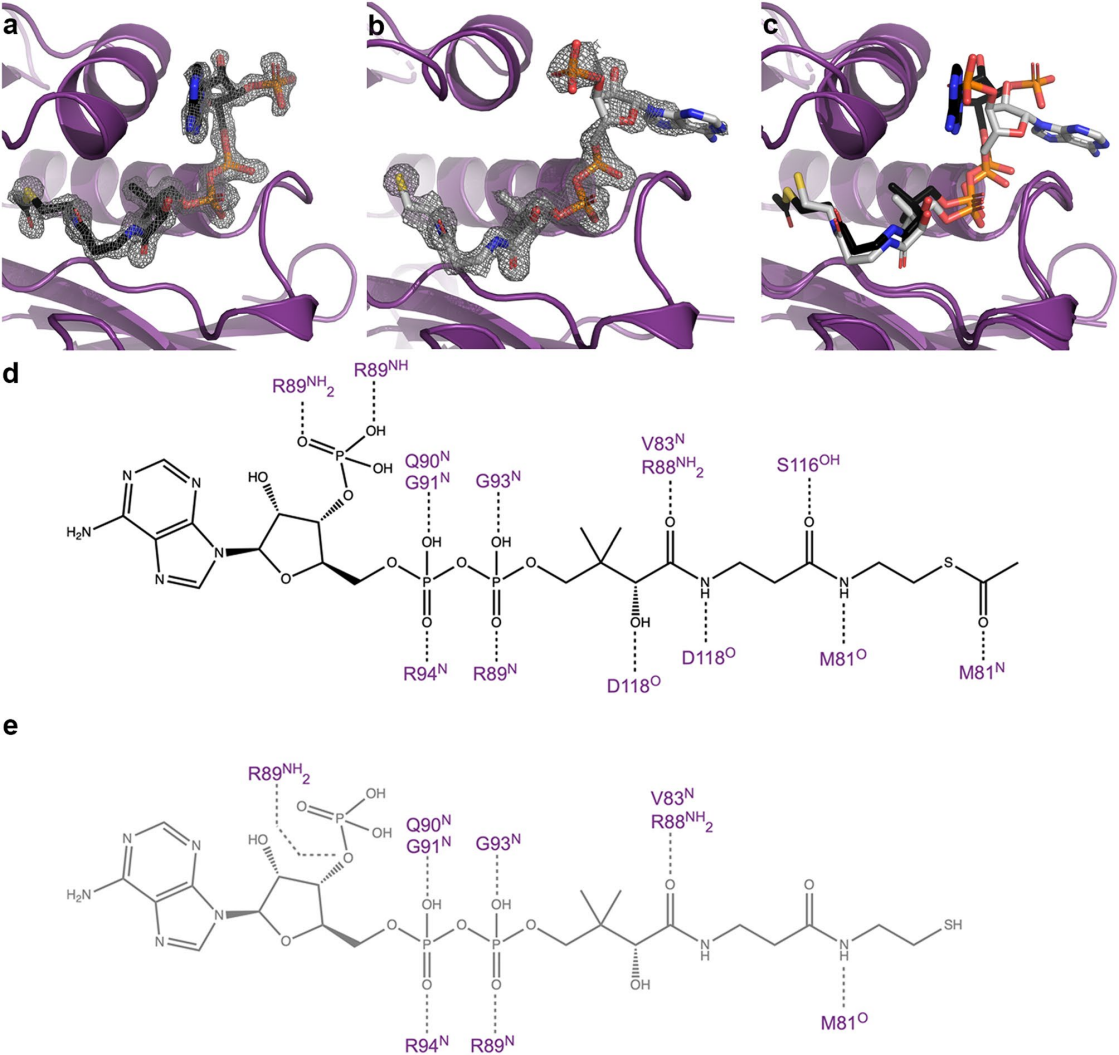
Interactions of Acetyl-CoA and CoA to AAC(2′)-Ia. Panels (**a**) and (**b**) depict representative Fo − Fc discovery maps for ligands acetyl-CoA and CoA from complex structures with amikacin and tobramycin, respectively, at a contour level of 3σ. (**c**) Overlay of AAC(2′)-Ia bound to acetyl-CoA and CoA, same color scheme as panels (**a**) and (**b**). Hydrogen bond interactions between AAC(2′)-Ia and (**d**) acetyl-CoA (black), (**e**) CoA (grey).

The CoA binding pocket of AAC(2′)-Ia has not previously been described. The discovery maps for each of the AAC(2′)-Ia crystal structures allow us to unambiguously place acetyl-CoA and CoA within the enzyme’s active site (Fig. 2a,b). The crystal structure of AAC(2′)-Ia in complex with acetyl-CoA and amikacin, a non-substrate aminoglycoside, is allowing us to capture the enzyme in a pre-catalysis state. Meanwhile, the crystal structures of AAC(2′)-Ia in complex with CoA and acetylated aminoglycosides describes the enzyme in three product-bound states. While there are a large number of flexible basic residues in the CoA and acetyl-CoA binding regions, most of the interactions within the binding sites observed in each monomer are maintained. In the pre-catalyzed state, the adenine rings of acetyl-CoA are sandwiched in between two flexible loops, with residue Arg89 on one side and Lys121 on the other; however, this portion does not form any inter- or intramolecular hydrogen bonds. The phosphoryl-group of the 3′-phosphorylated adenine diphosphate (ADP) moiety forms hydrogen bond interactions with Arg89, while the ribose ring is void of interactions with the enzyme (Fig. 2d). The α- and β-phosphates of this same moiety form backbone hydrogen bond interactions with Gln90, Gly91 and Arg94, as well as Arg89 and Gly93, respectively (Fig. 2d). In the pantothenic acid moiety, the hydroxyl group of the carboxylic acid hydrogen bonds with Asp118, while the oxygen can interact with Arg88 or Val83. The second oxygen of this moiety interacts with Ser116 (Fig. 2d). The amide groups of acetyl-CoA form hydrogen bonds with Asp118 and the backbone oxygen of Met81, respectively (Fig. 2d). Finally, the oxygen group of the acetyl portion forms a hydrogen bond with the backbone amide of Met81 (Fig. 2d).

In the three product-bound states of AAC(2′)-Ia, the pantothenic acid moiety and the α- and β-phosphates of the 3′-phosphorylated ADP moiety of CoA remain in a similar conformation to that of acetyl-CoA (Fig. 2c). The hydrogen bond interactions remain similar, except towards the tail end of the CoA molecule, where the pantothenic acid moiety forms fewer interactions with the enzyme. (Fig. 2e). The most apparent difference between acetyl-CoA and CoA binding to the pre-catalyzed-state and the product-bound state is the positioning of the 3′-adenosine moiety. In the product-bound state, this portion of CoA flips roughly 270° towards a solvent-exposed portion of the enzyme (Fig. 2c). The flipping of the 3′-adenosine moiety is likely due to the solvent-exposed nature of the active site, as well as space group differences between the pre-catalyzed and product-bound states. Clashes from molecules in the asymmetric unit likely inhibit this moiety of acetyl-CoA from adopting the same conformation seen in the pre-catalyzed state. However, the flexibility of the 3′-adenosine moiety does not introduce notable downstream changes in the binding of the acetyl-CoA molecule. As previously noted, the ribose and adenine rings do not interact with the enzyme. Instead, the adenine rings are now adjacent to a loop containing residues Arg89-Gly91.

### Structural basis for aminoglycoside binding to AAC(2′)-Ia

**Figure 3:**
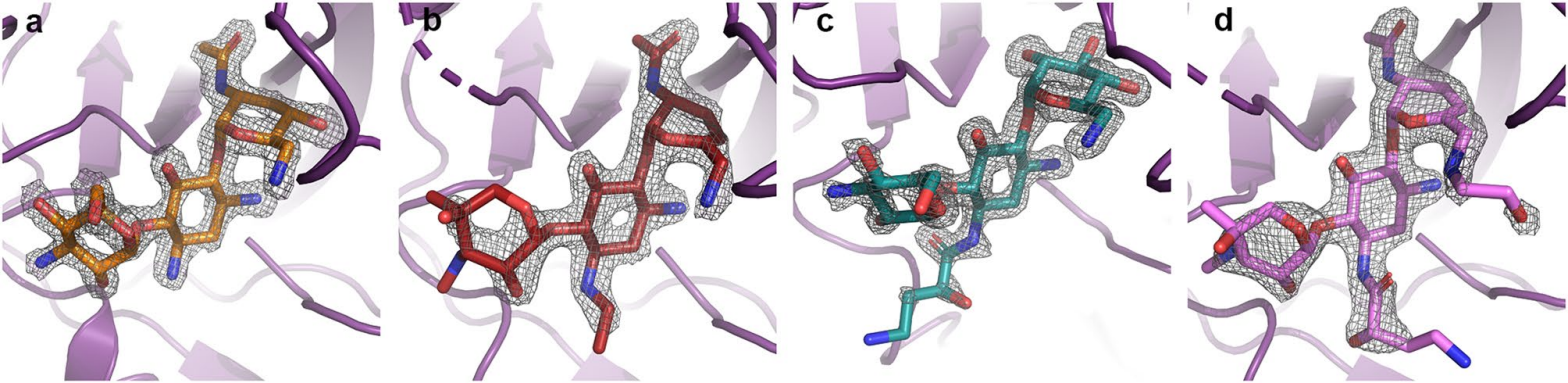
Binding of naturally occurring, semi-synthetic and next-generation aminoglycosides to AAC(2′)-Ia. Depicted in panels (**a**), (**b**), (**c**), and (**d**) are the Fo − Fc discovery maps for ligands tobramycin, netilmicin, amikacin, and plazomicin, respectively, at a contour level of 3σ. The plazomicin structure is presented here for comparison^18^. The enzyme is colored purple and the aminoglycosides tobramycin, netilmicin, amikacin, and plazomicin are colored in orange, red, teal, and violet, respectively.

**Figure 4:**
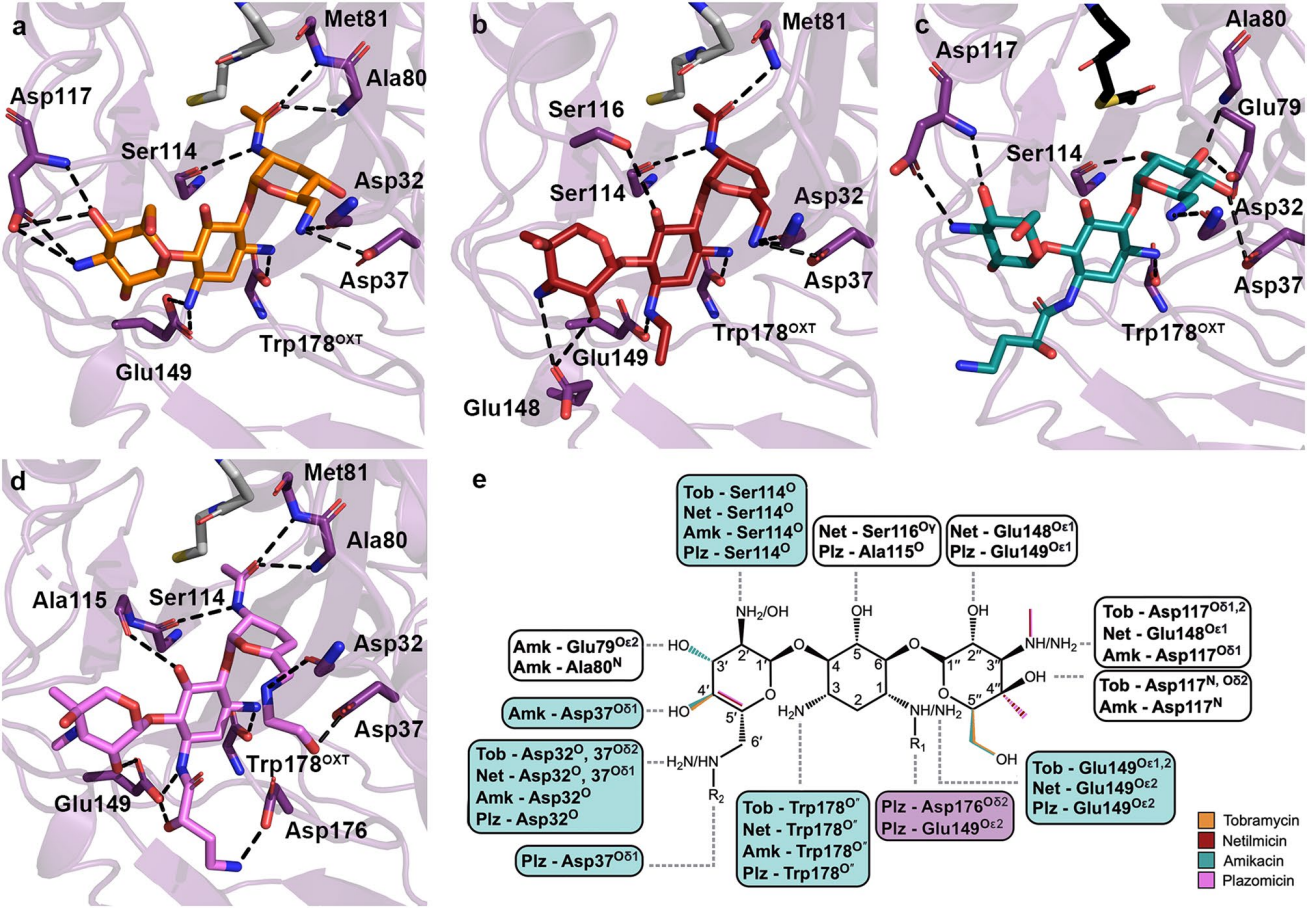
Interactions of naturally occurring, semi-synthetic aminoglycosides, and plazomicin to AAC(2′)-Ia. Depicted in panels (**a**), (**b**), (**c**), and (**d**) are the hydrogen bond interactions for acetylated tobramycin, acetylated netilmicin, amikacin, and acetylated plazomicin, respectively. Aminoglycosides are colored as in Fig. 3. The acetylated or non-acetylated β-mercapto-ethylamine moiety of acetyl-CoA and CoA ligands are shown for perspective, and are colored in either black or grey, respectively.(**e**) A combined chemical structure for all four aminoglycosides is shown Features unique to each aminoglycoside are highlighted based on the previous panel’s color scheme. Hydrogen bond interactions are depicted in boxes. Blue boxes indicate the four conserved sites involved in critical hydrogen bond interactions, and white boxes indicate interactions that occur less frequently. The box for the residues critical for plazomicin hydrogen bonding is colored in purple.

The discovery maps for each of the AAC(2′)-Ia crystal structures also allowed us to unambiguously place the aminoglycosides within the enzyme’s active site (Fig. 3a-d). The kinetic parameters for each aminoglycoside substrate are highlighted in Table 2. The structural basis for acetylated-tobramycin is similar to that of a previously reported acetylated-gentamicin-bound structure of AAC(2′)-Ia^30^. While the aminoglycoside portion of acetylated-gentamicin only hydrogen bonds with AAC(2′)-Ia via three residues, the tobramycin moiety can interact with up to 7 residues. In identical fashion to gentamicin, the central ring of tobramycin is anchored by two hydrogen bond interactions, N-1 with Glu149 and N-3 with the C-terminal carboxylate of Trp178 (Fig. 4a)^30^. Additionally, both aminoglycosides interact with the backbone carbonyl of Ser114 at their site of modification, N-2′ (Fig. 4a)^30^. Aside from these interactions, the prime-ring of tobramycin also interacts with the backbone carbonyl of Asp32 and the side chain of Asp37 at the N-6′ position (Fig. 4a). At the double-prime ring, tobramycin interacts with the enzyme at its N-4′′ and O-5′′ via hydrogen bond with Asp117 (Fig. 4a). The tobramycin molecule in chain B also forms an additional hydrogen bond interaction between its O-2′′ and Glu148 of AAC(2′)-Ia. The acetyl group modification of the molecule interacts with the enzyme by way of the backbone amine of Met81 (Fig. 4a). The acetylated tobramycin in chain A also interacts with the backbone amine of the adjacent residue, Ala80 (Fig. 4a).

**Table 2:** Kinetic parameters for AAC(2′)-Ia from *P. stuartii* and AAC(2′)-I from *D. wulumuqiensis* against aminoglycosides.

Aminoglycoside	k_cat_ (s^−1^)	K_M_ (μM)	k_cat_/K_M_ (s^−1^ μM^−1^)
**AAC(2′)-Ia from** ***P. stuartii***			
Tobramycin	5.04 ± 0.17	14.5 ± 1.7	0.348 ± 0.051
Gentamicin	2.75 ± 0.10	5.7 ± 0.9	0.482 ± 0.092
Netilmicin	6.05 ± 0.13	19.9 ± 1.3	0.304 ± 0.026
Plazomicin	1.42 ± 0.05	19.1 ± 2.5	0.074 ± 0.012
**AAC(2′)-I from** ***D. wulumuqiensis***			
Plazomicin	2.38 ± 0.14	24.4 ± 4.7	0.097 ± 0.025

AAC(2′)-Ia is capable of modifying three semi-synthetic aminoglycosides i.e., dibekacin, netilmicin, and plazomicin. Netilmicin is a semi-synthetic derivative of sisomicin, an aminoglycoside that is structurally similar and has comparable bacterial targets to gentamicin. Just as for gentamicin, AAC(2′)-Ia utilizes the same three residues, Ser114, Glu149, and Trp178, for hydrogen bonding (Fig. 4b). However, unlike gentamicin, netilmicin’s N-6′ can form hydrogen bonds with both Asp32 and 37 (Fig. 4b). This is due to a slight shift in the N-6′ group’s positioning. Additionally, Glu148 in chain A flips in towards the active site to interact with the 2′′-OH and 3′′-NH segments of netilmicin (Fig. 4b). Finally, the presence of a bulky ethyl substituent at N-1 does not cause any structural rearrangements of the enzyme, since this portion of the active site is readily solvent accessible.

Amikacin, a structural derivative of kanamycin, incorporates an (S)-HABA group at its N-1 position, but lacks an amine group at its N-2′ position, making it impervious to the effects of AAC(2′)-Ia (K_i_ = 87 ± 10 µM). Although plazomicin is the only aminoglycoside substrate of AAC(2′)-Ia with an N-1 (S)-HABA group, understanding how another N-1 substituted aminoglycoside binds to this enzyme can provide additional reasoning behind this enzyme’s specificity. Amikacin’s interactions are similar to those between the enzyme and its natural substrates (Fig. 4c). Although interactions are maintained, especially at the prime ring, the slight movement of residues around the active site in the amikacin-bound structure causes deviations in the positioning of the central and double-prime rings of the aminoglycoside. Additionally, the N-1 substitution does not form any hydrogen bond interactions with the enzyme, and instead, residues Glu148 and Glu149 shift to make it so the enzyme can accommodate the (S)-HABA group in a conformation away from the active site (Fig. 4c). This also shifts the binding of the double-prime ring to interact similarly to that of the acetylated-tobramycin-bound structure, as they both have similar chemical groups for this ring.

Comparing netilmicin and amikacin binding to AAC(2′)-Ia with how plazomicin interacts with the enzyme reveals similarities, as well as intriguing differences. Plazomicin, like netilmicin, is a semi-synthetic derivative of sisomicin. As such, its binding is essentially identical, except at its N-1 and N-6′ substituents, where due to chemical differences in the extensions, these two next-generation aminoglycosides form differing interactions (Fig. 4d). Surprisingly, when comparing amikacin with plazomicin, different interactions are noted at the N-1 site. As mentioned above, both amikacin and plazomicin incorporate the (S)-HABA group at the N-1 position; however, while this moiety in amikacin does not form any specific interactions with the enzyme, the (S)-HABA tail of plazomicin forms hydrogen bonds with Glu149 and Asp176 (Fig. 4d).

### Substrate specificity of the AAC(2′) enzyme class

Applying a phylogenetic analysis can provide an understanding of other potential AAC(2′) enzymes that can bind and modify plazomicin, and ultimately spread resistance to this aminoglycoside. A search of sequence databases using the five members of the AAC(2′)-I enzyme class identified 56 additional homologues from unique bacterial species filtered from 5000 psi-BLAST result sequences (1000 sequences from each psi-BLAST search). The identified sequences had at least a 23% sequence identity to the original query sequences (AAC(2′)-Ia—AAC(2′)-Ie). Based on the substrate-binding analysis, we identified three key residues involved in AAC(2′)-Ia’s aminoglycoside specificity; Asp37, Glu149, and Trp178, and one additional residue, Asp176, required for plazomicin specificity (Fig. 4e). Asp32 and Ser114 are also important for substrate binding in all four structures; however, they were excluded from the conservation analysis since they interact with aminoglycosides using their backbone atoms (Fig. 4e). Although Trp178 also employs its backbone oxygen to interact with aminoglycosides, this is an unusual interaction. Trp178 is the C-terminal residue, and it is the carboxyl group that forms a hydrogen bond and charge interaction with aminoglycosides. Ordinarily, protein termini are inherently flexible, but in AAC(2′)-Ia, the Trp178 side-chain forms hydrogen bond and van der Waals interactions with the rest of the enzyme, pinning the terminal carboxyl group in a specific location and orientation. Therefore, our sequence analysis includes an inherent assessment of Trp178′s residue conservation simultaneously with its positioning as the last residue in the sequence. It is worthwhile noting that interactions between the enzyme’s carboxy-terminus and aminoglycosides have also been observed in other AMEs^12,19,31^.

**Figure 5:**
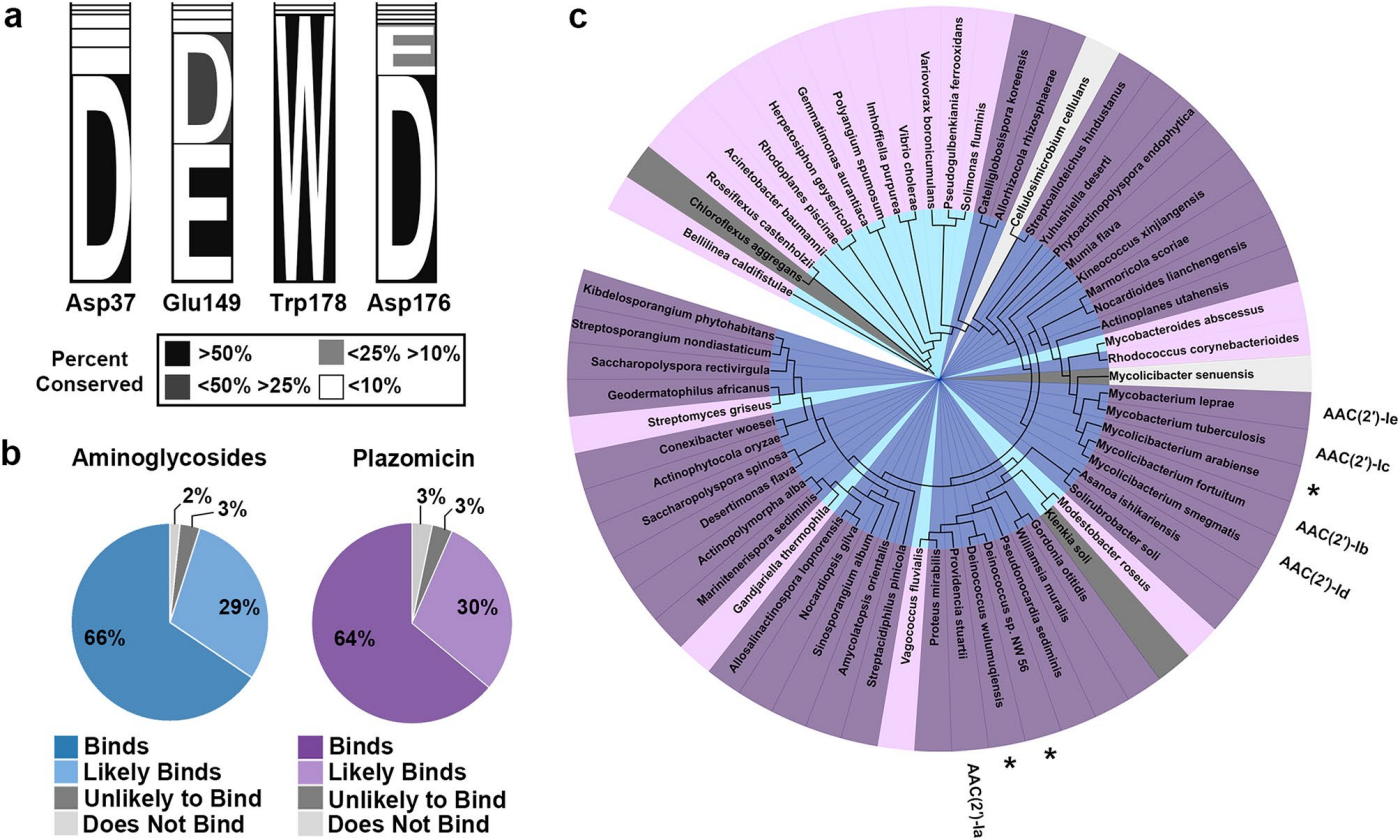
Phylogenetic analysis of aminoglycoside (2′)-N-acetyltransferase substrate specificity. (**a**) Percent conservation of key residues involved in aminoglycoside and plazomicin binding, where conservation is colored according to the legend. (**b**) Predicted classification of 61 identified sequences as binders, likely binders, unlikely binders, and non-binders for aminoglycosides or plazomicin, colored according to the legend. (**c**) Phylogenetic tree of identified sequences colored as in panel (**b**), with aminoglycosides’ coloring in the center, and plazomicin coloring in the outer circle. The original five query sequences are labelled, and plasmid-encoded sequences are highlighted with an asterisk (*).

Sequence alignment of all 61 unique sequences (5 query sequences and 56 identified homologues) shows that the four residues involved in aminoglycoside binding are highly conserved (Fig. 5a). Asp37, Glu149, Trp178, and Asp176 are conserved 74, 88, 95, and 92% of the time, respectively, where conservation percentages for Glu149 and Asp176 include instances of both aspartic and glutamic acid (Fig. 5a). Interestingly, although Asp32 utilizes its backbone oxygen for binding, it is still conserved 75% of the time.

The predicted ability of the 61 enzymes to bind to either aminoglycosides or next-generation aminoglycoside plazomicin was assessed based on the number of conserved binding residues (Fig. 5b,c). Note, the prediction of aminoglycoside binding is based on enzymes whose substrate specificity has been described [AAC(2′)-Ia-e], however, the prediction of plazomicin binding is based solely on data from AAC(2′)-Ia. The assembly of enzymes were classified as either non-binders, unlikely binders, likely binders, or binders of aminoglycosides if 0, 1, 2, or 3 of the binding residues (Asp37, Glu149, Trp178) were observed in a sequence, respectively. The same classification was made for plazomicin if 0–1, 2, 3, or 4 of the binding residues (Asp37, Glu149, Trp178, Asp176) were observed in a sequence, respectively (Fig. 5b). For aminoglycosides, it was assessed that 2, 3, 29, and 66% of enzymes would be non-binders, unlikely binders, likely binders, and binders, respectively, reflective of the extensive conservation observed in the substrate binding site (Fig. 5a,b). Similarly, for plazomicin, it was predicted that 3, 3, 30, and 64% of enzymes would follow the same binding pattern, respectively (Fig. 5b). It is meaningful to note that only three enzymes change their classification on their ability to bind either aminoglycosides or next-generation aminoglycoside plazomicin (Fig. 5c). Sequence alignment of a subset of sequences from each of the binding classifications are provided in Supplementary Fig. S2 online.

**Figure 6:**
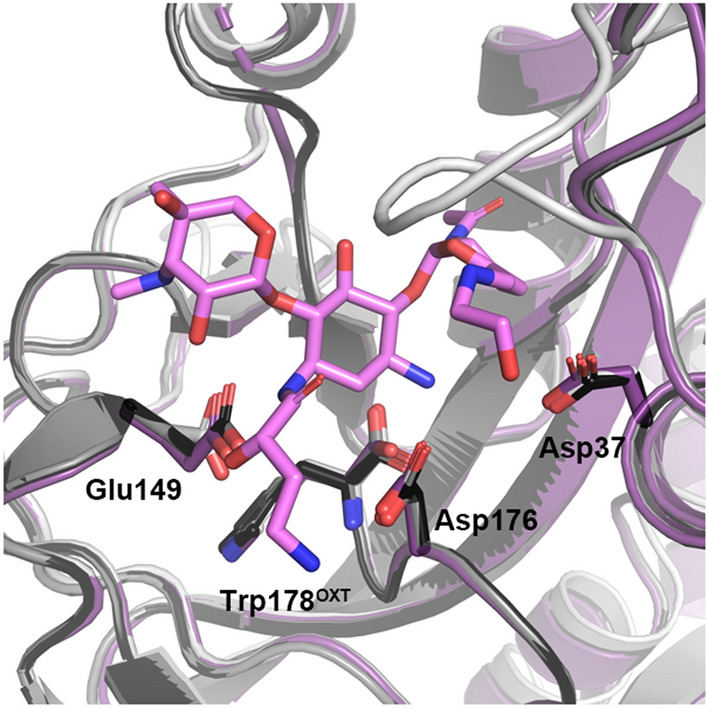
Active site overlay and conserved residues of AAC(2′)-Ia and modeled enzymes. AAC(2′)-Ia from *P. stuartii* is colored in purple, while the models are colored black (*D. sp. NW-56*), grey (*D. wulumuqiensis*) and light grey (*M. arabiense*). Plazomicin is depicted in stick representation and colored in violet.

The majority of the sequences found are chromosomally encoded, partly reflecting the contents of sequence databases; however, three of the sequences found are plasmid-encoded. These three sequences are found in the following bacterial species: *Mycolicibacterium arabiense*, *Deinococcus wulumuqiensis*, and *Deinococcus* sp.* NW-56* (RefSeq Reference: WP_163924889.1, WP_114673790.1, WP_104992197.1) (Fig. 5c)^32,33^. Each of these sequences shares a 33.5, 42.0, and 43.5% sequence identity, and an 84, 98, and 99% sequence coverage with AAC(2′)-Ia from *P. stuartii*, respectively, where all four binding residues are conserved. A sequence alignment of all three sequences is provided in Supplementary Fig. S2 online. Models of these three enzymes show their aminoglycoside binding pocket can readily bind plazomicin (Fig. 6). To test this, we expressed and purified the *D. wulumuqiensis* homologue and examined its kinetic parameters against plazomicin. As predicted, AAC(2′)-I from *D. wulumuqiensis* is found to have very similar kinetic parameters against plazomicin compared with AAC(2′)-Ia from *P. stuartii* (Table 2).

## Discussion

Plazomicin has shown activity against methicillin-resistant *Staphylococcus aureus* and multi-drug resistant *Escherichia coli*, *Klebsiella pneumonia*, and *Enterobacter* spp., and it is currently approved for the treatment of complicated urinary tract infections and pyelonephritis^34,35^. Plazomicin’s effectiveness stems from the incorporation of two chemical groups at the N-1 and N-6′ positions of its aminoglycoside precursor, sisomicin^36^. It is understood that elevated activity against plazomicin is exclusive to AAC(2′)-Ia from *P. stuartii*, whereas van der Waals strain prevents clinically widespread resistance factors such as ANT(2′′), APH(2′′), and AAC(6′) from binding this antibiotic^11,36^. We provide here a detailed structural analysis of an aminoglycoside modifying enzyme that can alter an aminoglycoside with several different semi-synthetic additions. Our investigation presents an understanding of why AAC(2′)-Ia has the ability to accommodate the N-1 (S)-HABA or ethyl groups and the N-6′ hydroxy-ethyl group. Comparison of three crystal structures presented here, together with our recently reported plazomicin complex structure^18^, allows us to inspect structural differences in active site binding at both these positions.

**Figure 7:**
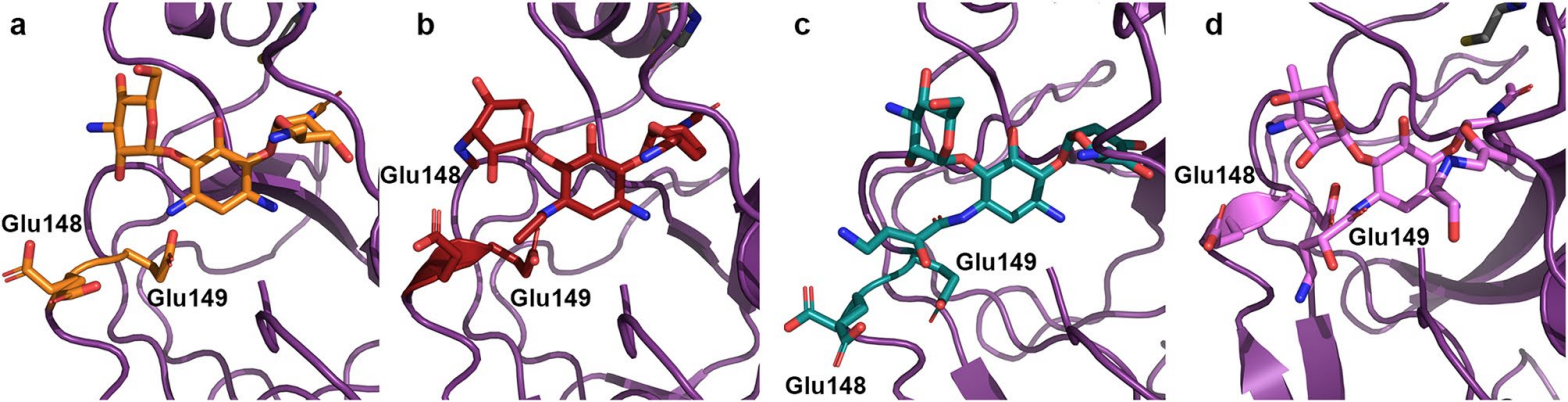
AAC(2′)-Ia residues responsible for aminoglycoside N-1 (S)-HABA group accommodation. Residues are colored according to the corresponding aminoglycoside; (**a**) tobramycin, (**b**) netilmicin, (**c**) amikacin, and (**d**) plazomicin. Colors are in accordance with the schemes presented in Figs. 3 and 4.

First, at the N-1 binding site, the accommodation of the (S)-HABA or ethyl groups is due to the flexibility of either residue Glu148 or Glu149. Our structures show that Glu148 can adopt two conformations (Fig. 7). The difference in the positioning of this residue is dependent on how the enzyme harbours the N-1 expansion. In the tobramycin-bound structure, there is no chemical addition at the N-1 amine, and therefore Glu148 can adopt either conformation (Fig. 7a). In the netilmicin bound structure, the ethyl addition at N-1 sits perpendicular to the aminoglycoside plane, requiring Glu148 to flip away from the active site (Fig. 7b). The plazomicin-bound structure sees the N-1 (S)-HABA adopting a similar conformation to the ethyl addition of netilmicin, where the (S)-HABA group first protrudes perpendicularly to the aminoglycoside plane, and then proceeds in a downward fashion (Fig. 7b,d). The amikacin-bound structure shows that the enzyme is also capable of adapting the (S)-HABA moiety in a second conformation. This second conformation, as displayed by amikacin, exhibits that the N-1 tail can also sit parallel to the aminoglycoside plane (Fig. 7c). The adoption of this conformation is based on the movement of residue Glu149 (Fig. 7c). As opposed to the other three structures, this residue flips away from the aminoglycoside-binding site to accommodate this bulky substituent.

Second, the N-6′ binding site consists of two residues, Asp32 and 37. With respect to aminoglycoside binding, accommodation of this chemical extension requires no conformational change when comparing binding to aminoglycosides void of this moiety. Moreover, interaction with these two residues is important for prime-ring binding in all four aminoglycoside-bound structures. It is noteworthy that the N-6′ hydrogen bond donor moiety of plazomicin has been implicated in being crucial for specificity for the bacterial ribosome over the eukaryotic homologue^18,37,38^.

Our analysis in Fig. 5 outlines the conservation of residues in the aminoglycoside/ plazomicin binding pocket across AAC(2′)-I enzymes. These residues are highly conserved, and our analysis suggests that 95% of the homologues that have been identified would be likely or capable of binding aminoglycosides (Fig. 5b,c). From these identified homologues, 98% of them are predicted to retain their ability to bind plazomicin (Fig. 5b,c). This result is troublesome as it reveals that plazomicin resistance is likely not isolated to a single bacterial species. Moreover, based on binding residue conservation, it is likely that the ability to chemically detoxify plazomicin is an innate feature of AAC(2′)-I enzymes.

Our analysis also provides an understanding of the evolutionary pathway for the AAC(2′)-I enzyme subtypes. Our survey of public sequence databases queried against five chromosomally encoded sequences from *P. stuartii* (AAC(2′)-Ia), *M. fortuitum* (AAC(2′)-Ib), *M. tuberculosis* (AAC(2′)-Ic), *M. smegmatis* (AAC(2′)-Id), and *M. leprae* (AAC(2′)-Ie) have allowed us to identify homologues from other species that are aminoglycoside and plazomicin binders^39–42^. While the results from this investigation show that the majority of homologues remain chromosomally encoded, three homologues were found to be plasmid-encoded (Fig. 5c). Additionally, these sequences have high similarity and identity to AAC(2′)-Ia, with all plazomicin binding residues being conserved (Fig. 6), where the *D. wulumuqiensis* homologue of AAC(2′)-I was found to have comparable kinetic parameters to AAC(2′)-Ia from *P. stuartii* against plazomicin (Table 2). This finding is cause for alarm as the ability of transposable elements to disseminate quickly allows resistance to spread in pathogens of medical interest^6^. Moreover, this reinforces the urgency of preserving plazomicin as a viable treatment option. Currently, plazomicin is on the WHO’s list of essential medicines, and is part of the reserve group of antibiotics, for use only against infections that are suspected to be caused by multidrug-resistant organisms. Although resistance is not yet widespread, the identification of sequences which are likely capable of binding plazomicin on mobile elements indicates that the spread of resistance is no longer theoretical, it is inevitable. This result, combined with the ability of AAC(2′)-Ia to bind N-1-substituted aminoglycosides in different conformations, enforces the need for next-generation aminoglycosides with a novel or additional chemical substituent in order to curb resistance to antibiotics of last resort.

## Methods

### Cloning

#### AAC(2′)-Ia from Providencia stuartii construct with non-cleavable C-terminal HIS-tag

The AAC(2′)-Ia gene from *Providencia stuartii* was synthesized and subcloned into the pET-15b expression vector between the NdeI and XhoI restriction sites with a non-cleavable C-terminal HIS-tag. From here forward in the methods section, we refer to this construct as AAC(2′)-Ia_HIS_, where HIS designates the non-cleavable HIS-tag. The DNA sequence was verified using the BioBasic Inc. gene synthesis service. The resulting vector was used to transform *E. coli* BL21(DE3) cells.

#### AAC(2′)-Ia from Providencia stuartii construct with cleavable N-terminal HIS-tag

A second AAC(2′)-Ia construct from *Providencia stuartii* was synthesized and subcloned into the pET-15b expression vector between the NdeI and BamHI restriction sites with an N-terminal HIS-tag followed by a thrombin cleavage site. From here forward in the methods section, we simply refer to this construct as AAC(2′)-Ia. The DNA sequence was verified using the BioBasic Inc. gene synthesis service. The resulting vector was used to transform *E. coli* BL21(DE3) cells.

#### AAC(2′)-I from Deinococcus wulumuqiensis

The AAC(2′)-I homologue from *Deinococcus wulumuqiensis* (RefSeq reference: WP_114673790.1) was synthesized and subcloned into the pET-15b expression vector between NdeI and BamHI restrictions sites with an N-terminal HIS-tag. From here forward in the methods section, we refer to this construct as AAC(2′)-I. The DNA sequence was verified using the BioBasic Inc. gene synthesis service. The resulting vector was used to transform LOBSTR *E. coli* cells^43^.

### Expression

Protein expression of the *P. stuartii and D. wulumuqiensis* constructs was carried out using the Studier method for auto-induction, as previously described^11,44^. Cells were harvested by centrifugation at 6000 g for 15 min at 4 °C and resuspended in 40 mL of lysis buffer. Lysis buffer for AAC(2′)-Ia_HIS_ and AAC(2′)-Ia constructs from *P. stuartii* consisted of 50 mM TRIS–HCl, pH 8.0, 200 mM NaCl, 10 mM β-mercaptoethanol, 10% (v/v) glycerol, and one EDTA-free protease inhibitor tablet (Roche); while lysis buffer for AAC(2′)-I from *D. wulumuqiensis* consisted of 50 mM bicine, pH 9.0, 300 mM NaCl, and 10 mM imidazole. Cells were lysed by sonication, and cell debris was subsequently removed by centrifugation at 50,000 g for 30 min at 4 °C. The supernatant was further clarified by filtration through a 0.22 μm syringe-driven filter.

### Purification

#### *Affinity purification of AAC(2′)-Ia*_*HIS*_* and AAC(2′)-Ia*

The same procedure for affinity purification was followed for the AAC(2′)-Ia_HIS_ and AAC(2′)-Ia constructs. Briefly, the resulting clarified material was applied on a 26 mm i.d. × 50 mm Ni-IDA-Sepharose column, equilibrated in 50 mM TRIS–HCl, pH 8.0, 200 mM NaCl, 10 mM β-mercaptoethanol, and 10% (v/v) glycerol; then eluted stepwise with starting buffer supplemented with 150 mM imidazole.

#### *AAC(2′)-Ia*_*HIS*_

Following affinity purification, fractions containing AAC(2′)-Ia_HIS_ were identified by SDS-PAGE and pooled. The pool was desalted on HiPrep 26/10 Desalting column (GE), equilibrated in 25 mM BIS–TRIS propane pH 7.5, 10 mM β-mercaptoethanol, and 10% (v/v) glycerol. The desalted material was applied on DEAE Sepharose FF 26 mm i.d. × 140 mm column, equilibrated in the identical buffer, and eluted with a 0–400 mM NaCl gradient over 16 column volumes. Peak fractions from the DEAE column were pooled, and buffer exchange was performed on the same desalting column, equilibrated in the final storage buffer consisting of 10 mM HEPES, pH 6.6, and 1 mM TRIS (2-carboxyethyl) phosphine hydrochloride (TCEP). AAC(2′)-Ia_HIS_ was then concentrated to 10 mg mL^−1^ and stored at 4 °C.

#### AAC(2′)-Ia

Following affinity purification, fractions containing AAC(2′)-Ia were identified by SDS-PAGE and pooled. 50 µL of 1-unit µL^−1^ thrombin was added to the pool and incubated overnight at 22 °C to remove the N-terminal HIS-tag. The pool was then applied on a HiTrap Benzamidine FF column (GE), equilibrated in 50 mM TRIS–HCl, pH 8.0, 200 mM NaCl, 10 mM β-mercaptoethanol, and 10% (v/v) glycerol, to remove thrombin from the AAC(2′)-Ia sample. AAC(2′)-Ia fractions were subsequently desalted on HiPrep 26/10 Desalting column (GE) and applied on the DEAE Sepharose FF 26 mm i.d. × 140 mm column as described for AAC(2′)-Ia_HIS._ AAC(2′)-Ia was then concentrated and stored, as above.

#### AAC(2′)-I Deinococcus wulumuqiensis

Fractions containing AAC(2′)-I from *D. wulumuqiensis* were subjected to affinity purification, where the resulting clarified material was applied to a 5 mL Ni–NTA Superflow Cartridge (Qiagen), equilibrated in 50 mM bicine, pH 9.0, 300 mM NaCl, and 10 mM imidazole; then eluted over a 10–300 mM imidazole gradient over 10 column volumes. Despite the presence of a thrombin cleavage site, the HIS-Tag was not removed. Fractions containing AAC(2′)-I from *D. wulumuqiensis* were identified by SDS-PAGE and pooled. The pool was concentrated on an Amicon Ultra (3 kDa) and applied on a HiLoad Superdex 200 16/60 (GE) column equilibrated in 20 mM MES, pH 6.5, and 150 mM NaCl. Fractions from a single peak were pooled and concentrated, then stored at 4 °C.

### Crystallization

Optimized crystals of AAC(2′)-Ia_HIS_ were grown at 22 °C using the sitting-drop vapour diffusion method. Drops contained a 1:1 ratio of 10 mg mL^−1^ of AAC(2′)-Ia_HIS_ in the storage buffer supplemented with 10 mM acetyl-CoA and 10 mM kanamycin. Crystals of AAC(2′)-Ia_HIS_ in complex with CoA grew when the reservoir solution consisted of 2 M ammonium sulfate, 2% (v/v) PEG 400, and 0.1 M HEPES, pH, 7.5. Note, kanamycin was not present in the final structure due to the presence of the C-terminal HIS-tag in the active site.

All crystals of AAC(2′)-Ia were grown at 4 °C using the sitting-drop vapour diffusion method. Drops contained a 1:1 ratio of 10 mg mL^−1^ of AAC(2′)-Ia in storage buffer supplemented with 10 mM acetyl-CoA and 10 mM antibiotic. Crystals of AAC(2′)-Ia in complex with CoA and acetylated tobramycin grew when the reservoir solution consisted of 35% (v/v) 2-methyl-2,4-pentanediol (MPD) and 0.1 M sodium acetate, pH 4.5. Crystals of AAC(2′)-Ia in complex with CoA and acetylated netilmicin grew when the reservoir solution consisted of 0.2 M sodium thiocyanate and 40% MPD. Finally, crystals of AAC(2′)-Ia in complex with acetyl-CoA and amikacin grew when the reservoir solution consisted of 1 M sodium citrate, 0.2 M NaCl, and 0.1 M TRIS, pH 7.0.

### Data collection

Diffraction data for optimized crystals of the four complexes were collected on a Bruker D8 Discovery consisting of a METALJET source (liquid gallium) coupled with a PHOTON II CPAD detector mounted on a KAPPA goniometer. A ten-fold data collection strategy was calculated for all data sets using the PROTEUM3 software suite (Bruker).

### Structure solution and refinement

Datasets for all structures were processed using the *xia2* pipeline^45^, [*CCP4*^46^, *POINTLESS*^47^, *XDS*^48^]. The structure of AAC(2′)-Ia_HIS_ in complex with CoA was solved by molecular replacement using *Phaser*^49^, using a previously solved structure as the search model (PDB ID: 5US1)^30^. The data collection and refinement statistics for this structure are listed in Supplementary Table S1 online. The structure of AAC(2′)-Ia in complex with acetylated tobramycin and CoA was solved by molecular replacement using *Phaser*, with the CoA complex of AAC(2′)-Ia_HIS_ used as the search model. The structure of AAC(2′)-Ia in complex with amikacin and acetyl-CoA was solved using molecular replacement with the acetylated tobramycin-CoA complex of AAC(2′)-Ia as the search model using *Phaser*. Finally, the structure in complex with acetylated netilmicin and CoA was determined using Fourier synthesis performed by *phenix.refine*^50^ using the acetylated tobramycin-CoA complex stripped of all non-protein atoms. All structures were refined by iterative cycles of reciprocal-space refinement with *phenix.refine* and real-space refinement and model building in *Coot*^51^. The ligand restraints for CoA, acetyl-CoA, acetylated antibiotics, and amikacin were generated using *eLBOW*^52^. The data collection and final refinement statistics of the three aminoglycoside-bound models are listed in Table 1.

### Phylogenetic Analysis of Aminoglycoside 2′-Acetyltransferases

AAC(2′) sequences were identified using PSI-BLAST (Position-Specific Iterated BLAST)^53^, with five separate searches using AAC(2′)-Ia, b, c, d, and e as the respective query sequences (GenBank/RefSeq reference: AAA03550, AAC44793, ACT23293, WP_011726942, CAC32082) ^39–42^. The search was run using the reference proteins database (RefSeq), excluding models and uncultured sequences^54^. Search parameters included 1000 maximum target sequences with an expect threshold of 10. BLOSUM62 was used as the scoring matrix. The PSI-BLAST threshold was set at 0.005 and was run for five iterations for each query sequence. Final sequences for the phylogenetic tree were chosen from each search list based on sequence identity (> 23%), clinical prevalence, and relative e-value. The alignment of the final 61 sequences was generated in NGPhylogeny.fr using the MAFFT L-INS-I method^55,56^. NGPhylogeny was subsequently used to generate a maximum-likelihood phylogenetic tree using the PhyML algorithm^55,57^. The final phylogenetic tree was designed using iTOL (Interactive Tree of Life)^58^. Plasmid-encoded sequences were modelled using the SWISS-MODEL server^59–62^, with the AAC(2′)-Ia-acetylated plazomicin complex stripped of its ligands and water molecules used as the template.

### Plazomicin Synthesis

Synthesis of plazomicin was performed starting from commercially available sisomicin sulfate as recently reported^63^ in the modified version of the original report by Moser^15^.

### Kinetic assay of AAC(2′)-Ia from *Providencia stuartii*

The kinetic parameters of AAC(2′)-Ia from *P. stuartii* against a panel of aminoglycosides (tobramycin, gentamicin, netilmicin and plazomicin) were obtained using the ThermoFisher NanoDrop One^C^ Spectrometer. The acetylation of aminoglycosides was measured by a coupled assay where the formation of pyridine-4-thiolate can be detected at 324 nm^64,65^. The assays were performed in a 0.8 mL quartz cuvette (pathlength 1 cm), in a buffer containing 25 mM MES, pH 5.5, 100 mM NaCl, 500 µM 4,4′-dipyridyl disulfide (Aldrithiol-4, Sigma-Aldrich), 150 µM acetyl-CoA, and varying aminoglycoside concentrations (2.5 to 160 mM). The reaction was initiated by the addition of enzyme (0.5 µM, final concentration), where UV absorbance was measured over 10 min at 22 °C. Assays were run in triplicate, and data analysis was performed using the GraphPad5 software.

### Inhibition of amikacin towards of AAC(2′)-Ia from *Providencia stuartii*

Inhibition assays to determine the K_i_ of non-substrate amikacin were performed against tobramycin using the same protocol as described above. The kinetic properties were measured against 0, 20, 80, 160, and 320 µM of amikacin (k_cat_ = 5.63 ± 0.10 s^−1^; K_M_ = 12.7 ± 1.1 µM; K_cat_/K_M_ = 0.44 ± 0.05 s^−1^ µM^−1^). As expected, amikacin acted as a competitive inhibitor towards tobramycin (K_i_ = 87 ± 10 µM).

### Kinetic assay of AAC(2′)-I from *Deinococcus wulumuqiensis*

The kinetic properties of AAC(2′)-I from *D. wulumuqiensis* against plazomicin were measured using the same assay as AAC(2′)-Ia from *P. stuartii*, with minor adjustments to the protocol. The assays were performed in the same buffer, except at pH 6.0. The aminoglycoside concentration range was adjusted (6.26 to 100 µM). The final concentration of the enzyme in the reaction mix was 0.14 µM.

### Accession codes

AAC(2′)-Ia-C-terminal HIS-tag in complex with CoA (PDB ID: 7JZS).

AAC(2′)-Ia in complex with acetyl-CoA and amikacin (PDB ID: 6VTA).

AAC(2′)-Ia in complex with CoA and acetylated-tobramycin (PDB ID: 6VR2).

AAC(2′)-Ia in complex with CoA and acetylated-netilmicin (PDB ID: 6VR3).

AAC(2′)-Ia in complex with CoA and acetylated-plazomicin (PDB ID: 6VOU).

## Supplementary Information

Supplementary Information
