# Supplementary material for: Structural and phylogenetic analyses of resistance to next-generation aminoglycosides conferred by AAC(2′) enzymes
Source: Sci Rep. 2021 Jun 2;11:11614. doi: 10.1038/s41598-021-89446-3 (PMC8172861; doi:10.1038/s41598-021-89446-3)
Supplement: Supplementary file 1 — Supplementary Information [file 41598_2021_89446_MOESM1_ESM.pdf]

Supplementary Material

Structural and Phylogenetic Analyses of Resistance to Next-generation Aminoglycosides Conferred by AAC(2′) Enzymes

Angelia V. Bassenden[1,2], Linda Dumalo[1,2], Jaeok Park[1,2], Jonathan Blanchet[1,2], Krishnagopal Maiti[3], Dev P. Arya[3], and Albert M. Berghuis[1,2,4,*]

[1]Department of Biochemistry, McGill University, McIntyre Medical Building, 3655 Promenade Sir William Osler, Montréal, Québec, Canada, H3G 1Y6
[2]Centre de Recherche en Biologie Structurale, McGill University, Bellini Life Science Complex, 3649 Promenade Sir William Osler, Montréal, Québec, Canada, H3G 0B1
[3]Department of Chemistry, Clemson University, Clemson, South Carolina, 29634, United States
[4]Department of Microbiology and Immunology, McGill University, Duff Medical Building, 3775 University Street, Montréal, Québec, Canada, H3A 2B4

*albert.berghuis@mail.mcgill.ca

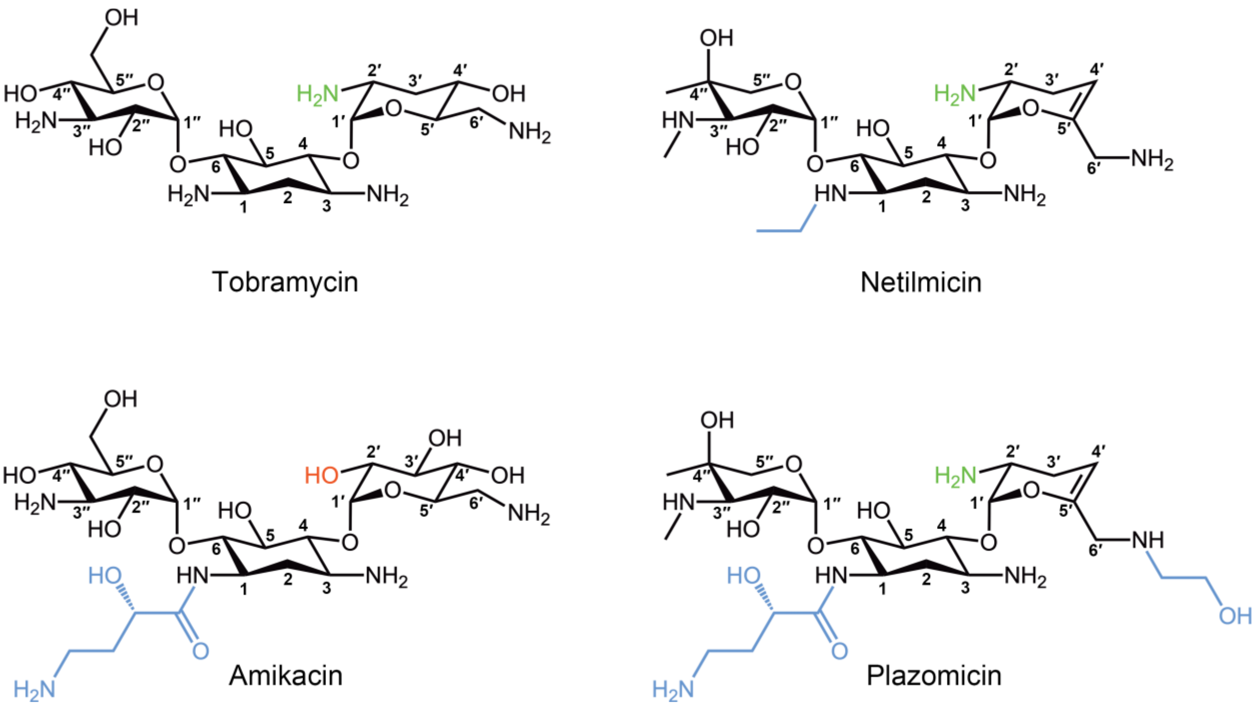

Figure S1: Panel of aminoglycosides used in crystallographic studies. Next-generation aminoglycoside features are colored in blue. Site of 2′-acetylation is colored in green, while 2′-hydroxyl of non-substrate, amikacin, is colored in red. Carbons are numbered as per their aminoglycoside ring, i.e., central ring, prime ring, or double-prime ring.

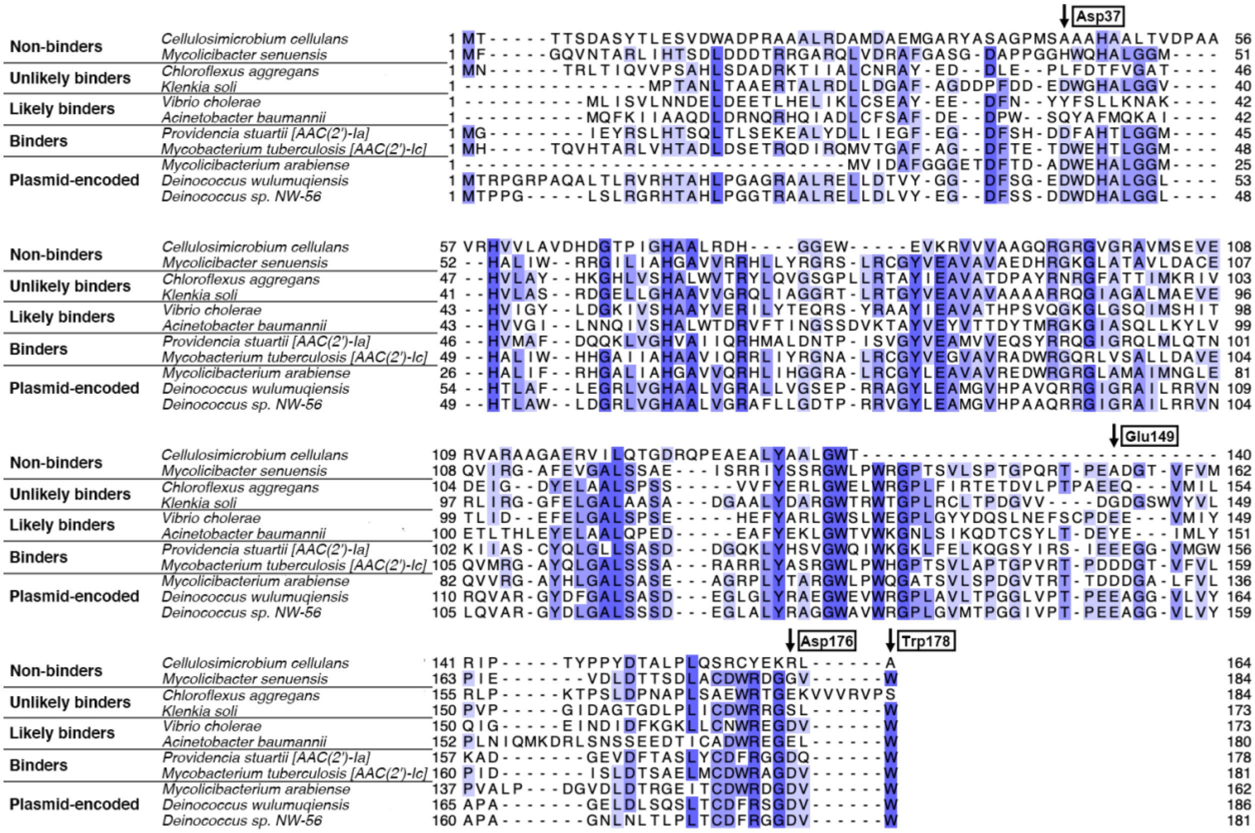

Figure S2: Alignment of a subset of sequences based on binding classification. The MAFFT sequence alignment highlights overall sequence identity in blue. Residues important for aminoglycoside binding are denoted with an arrow. Identified residues are numbered as per AAC(2′)-Ia from *Providencia stuartii*.

	**AAC(2′)-Ia$_{HIS}$ • CoA**
**Resolution range (Å)**	52.75-1.3 (1.346-1.3)
**Space group**	P 43 21 1
**Unit cell (Å, °)**	58.7 58.7 120.0
**Total reflections**	632665 (60458)
**Unique reflections**	52409(5117)
**Multiplicity**	12.1 (11.8)
**Completeness (%)**	100.0 (99.9)
**Mean I/sigma(I)**	28.1 (2.6)
**Wilson B-factor**	19.9
**R-merge**	0.045 (1.58)
**R-meas**	0.047 (1.65)
**$CC_{1/2}$**	1.00 (0.65)
**CC***	1.00 (0.89)
**Refinement Statistics**	
**R_{work}**	0.148 (0.206)
**R_{free}^{a}**	0.189 (0.234)
**Number of non-hydrogen atoms**	1982
**macromolecules**	1726
**ligands**	65
**water**	191
**Protein residues**	182
**RMS (bonds)**	0.030
**RMS (angles)**	2.4
**Ramachandran favored (%)**	94.4
**Ramachandran outliers (%)**	1.1
**Clashscore**	12.63
**Average B-factor**	29.9
**macromolecules**	28.0
**ligands**	38.0
**solvent**	44.3

Table S1: Data Collection and Refinement Statistics of AAC(2′)-Ia C-terminal His Complex. Statistics for the highest-resolution shell are shown in parentheses.

^{a} R_{free} was calculated by randomly omitting 10% of observed reflections from refinement.
